# Advancing palm genomics by developing a high-density battery of molecular markers for *Elaeis oleifera *for future downstream applications

**DOI:** 10.1186/1753-6561-8-S4-P96

**Published:** 2014-10-01

**Authors:** Alexandre Alonso Alves, Valquiria Martins Pereira, André Pereira Leão, Eduardo Fernandes Formigheri, Guy de Capdeville, Manoel Teixeira Souza Junior

**Affiliations:** 1 Embrapa Agroenergy, Parque Estação Biológica, Brasília/DF, 70770-901, Brazil; 2 Universidade Federal de Lavras - UFLA, Campus Universitário, Lavras, MG, CEP 37200-000, Brazil

## Background

Although (*Elaeis guineensis*) is planted on only 5% of the total world vegetable oil acreage, it accounts for more than 30% of vegetable oil produced worldwide [1]. In Brazil, the Federal Government launched a plan to boost Oil Palm production as a way to meet the biofuels market increasing demand for vegetable oil. Nevertheless, the Oil Palm expansion areas in Brazil coincide with the area of occurrence of bud rot [2], a major disease that is decimating plantations already established in the area. In a way to circumvent such problem, breeders are now using *E. oleifera *germplasm in Oil Palm breeding programs, generating inter-specific hybrids not only resistant to bud rot, but with higher unsaturated fatty acid content, lower height [3]. *E. oleifera*, however, lack the genomic resources currently available for Oil Palm [1], hampering many possible studies that could potentially help breeding. Based on the foregoing, we started a project to develop a large set of molecular markers for the species based on the DArTSeq platform [4].

## Methods

We collected 552 leaf samples from 206 accessions of the Embrapa *E. oleifera *germplasm collection. DNA was extracted and purified and sent for DArT-Pty for DArTSeq development. A combination of restriction enzymes (RE) was used to reduce the complexity of the samples that were later pooled sequenced in the Illumina´s HiSeq 2000 platform. After barcode sorting, sequences were trimmed and used to construct a reference sequence from all unique fragments. On average we obtained more than 2 million reads for each genotype. The individual reads were then aligned to the reference sequence and silico-DArTs scored binning nearly identical sequences so that the presence of an SNP would not hinder the scoring. Using the alignment of the reads to the reference sequence, SNP markers were also discovered based on a pipeline that uses bowtie as a component.

## Results and conclusions

We've obtained using the DArTSeq platform over 7.000 silico-DArTs markers. Once we applied a stringent selection criterion (Call Rate >0.90, MAF >0.05, and Q Value >2), 3.187 markers survived. Based on the alignment of the reads to the reference sequence we've also discovered 5.500 high-quality SNP loci, and other 1.950 lower-quality SNPs. This set of polymorphic markers may now be used for a series of downstream applications on palm genetics and genomics. For instance, we're using such markers to perform a large genetic diversity study of the Brazilian *E. oleifera *germplasm. As we are also sequencing the whole genome of the species [5], we plan to map the polymorphic reads to the reference genome assembly to assess the maker distribution on the genome and its co-localization with predicted gene models, as well as the physical distance between adjacent markers. The genomic position of the polymorphism is also expected to help the designing of probes for target-enrichment of polymorphic sites.
